# “The Box Has Become an Indispensable Part of My Life”: A Case Study of Victoria Cannabis Buyers Club and its Consumption Space

**DOI:** 10.1177/00914509231183147

**Published:** 2023-06-22

**Authors:** Marilou Gagnon, Alayna Payne, Zach Walsh, Adrian Guta, Carol Strike

**Affiliations:** 1 School of Nursing, 8205 University of Victoria, Victoria, British Columbia, Canada; 2 Canadian Institute for Substance Use Research, 8205 University of Victoria, Victoria, British Columbia, Canada; 3 Department of Psychology, 8166 University of British Columbia, Vancouver, British Columbia, Canada; 4 School of Social Work, 8637 University of Windsor, Windsor, Ontario, Canada; 5 Dalla Lana School of Public Health, 7938 University of Toronto, Toronto, Ontario, Canada

**Keywords:** cannabis, cannabis clubs, consumption space, legalization, smoking, therapeutic

## Abstract

Community-based models of cannabis cultivation, distribution, and consumption—such as cannabis clubs—have been documented across Europe, North America, South America, and New Zealand since the 1990s. For the most part, these models have a history of operating outside existing legislation and regulations. Jurisdictions that have legalized cannabis have approached community-based models in opposite ways (eliminate vs. regulate). Canada legalizing cannabis has resulted in more stringent enforcement and concerted efforts to close these models despite documented health and social benefits. This paper presents a case study of the Victoria Cannabis Buyers Club (VCBC) and its consumption space—The Box. We conducted a survey of VCBC members to explore four domains: demographics, cannabis consumption, access to and use of The Box, and the impact of its temporary closure due to COVID-19. From the survey data (*n* = 104), descriptive statistics were generated and three conceptual avenues were identified. The majority of respondents were 40 years old and older and identified as White (European descent) cisgendered men and women. The majority reported an income of $40,000 or less and a housing status that prevented them from smoking. Close to 75% of our sample consumed cannabis multidaily for therapeutic purposes primarily, but also for a mix of recreation, social, spiritual, and traditional healing purposes. Smoking was the preferred mode of consumption. Respondents accessed The Box daily or weekly. Reasons and benefits for using The Box fell into three categories: public health, harm reduction, and wellness perspectives. Conceptually, we found that The Box acted as a therapeutic space and offered a much-needed consumption space for smokers. We also identified a need to unpack the concept of safety. Overall, the survey reinforces the need for an equity-informed approach to community-based models and cannabis consumption spaces in Canada.

Community-based models of cannabis cultivation, distribution, and consumption have been documented across Europe, North America, South America, and New Zealand since the 1990s (Decorte et al., 2017; Jansseune et al., 2019; Pardal, 2016, 2020, 2023; Queriolo et al., 2016; Reiman, 2014). These models include cannabis clubs (including cannabis social clubs, cannabis buyers clubs, and cannabis compassion clubs), cannabis dispensaries, and cannabis distribution projects. All three models of cannabis clubs share similar characteristics in that they are nonprofit, driven by cannabis users, rely on a membership or intake system, and supply members with low-cost and high-quality cannabis for their personal use (Belackova et al., 2016; Belackova & Wilkins, 2018). Historically, cannabis clubs were developed to meet the needs of people who use cannabis for therapeutic and/or recreational purposes (Feldman & Mandel, 1998; Parés-Franquero et al., 2019). Cannabis dispensaries, on the other hand, are more akin to community pharmacies or storefront retailers. Many were introduced to help people access medical cannabis, whether authorized by a government-run medical cannabis program or not (Capler et al., 2017; Valleriani et al., 2020). It is important to note, however, that dispensaries and compassion clubs are sometimes used interchangeably in the Canadian literature. Cannabis distribution projects are more recent and unique to the Canadian context (St. Pierre et al., 2022; Valleriani et al., 2020). They provide cannabis products as safer alternatives to the unregulated poisoned opioid supply, without barriers and restrictions, and rely exclusively on grassroots-level donations and distribution efforts (St. Pierre et al., 2022; Valleriani et al., 2020).

Community-based models were born out of three types of policy problems. First, to address supply gaps and challenges created by prohibition, de facto decriminalization in the absence of legalization, and legalization (Decorte & Pardal, 2020). For people who use cannabis for therapeutic purposes (CTP), for instance, these models offer a middle-ground option between an underground market that does not meet their unique needs and government-run medical cannabis pathways that are overly cumbersome and restrictive (Valleriani, 2022). Second, to offer a range of cannabis products to people who use CTP and who have historically been excluded from and underserved by the government-run medical cannabis programs. For example, cannabis dispensaries and compassion clubs played a pivotal role in filling persistent gaps in the Canadian medical program, which relied heavily on physician gatekeeping and limited rather than expanded access to CTP (Belle-Isle et al., 2014; Capler et al., 2017; Capler & Bear, 2023; Fischer et al., 2015, 2020; Lucas, 2008, 2009, 2012; Ng et al., 2022; Valleriani, 2022; Walsh et al., 2013). Third, to implement a harm reduction and substitution tool in the context of an overdose crisis fueled by a highly toxic illicit opioid supply (Valleriani et al., 2022). Overall, community-based models share similar goals in that they seek to provide direct and low-threshold access to low-price and high-quality cannabis products while also offering education, support, and a sense of community (Decorte, 2015; Feldman & Mandel, 1998; Hathaway & Rossiter, 2007; Lucas, 2009).

Studies conducted on community-based models of cannabis cultivation, distribution, and consumption have documented several benefits. These models generate public health benefits by way of quality control, product information, membership or intake process (e.g., age), decreased public use, and promotion of responsible use (Belackova et al., 2016; Capler et al., 2017; Pardal, 2016; Parés-Franquero et al., 2019; Valleriani, 2022). They also increase access to education and guidance, which in turn, promotes informed decision-making and can reduce potential harms (Belackova et al., 2016; Capler & Bear, 2023; DeAngelo, 2015; Hathaway & Rossiter, 2007; Parés-Franquero et al., 2019; Subritzky, 2018). Moreover, these models have social benefits by creating opportunities for mutual aid, contributing to a sense of belonging, and increasing access to community resources and activities, as well as fostering a broader sense of healing, connectedness, and spirituality (Capler & Bear, 2023; DeAngelo, 2015; Feldman & Mandel, 1998; Hathaway & Rossiter, 2007; Lucas, 2009; Subritzky, 2018; Valleriani, 2022). Finally, recent studies suggest that community-based models of cannabis cultivation, distribution, and consumption have a role to play in scaling up cannabis substitution approaches amid the opioid overdose crisis by improving pain management, increasing rates of injection cessation, and contributing to improved treatment outcomes and adherence (Chayama et al., 2021; Lake et al., 2019; Lucas et al., 2021; Mok et al., 2021; Reddon et al., 2020; Socias et al., 2021; St. Pierre et al., 2022; Valleriani et al., 2020; Voon et al., 2018).

Community-based models of cannabis cultivation, distribution, and consumption tend to operate outside existing legislation and regulations (Decorte, 2015). These models have been historically “tolerated” by authorities and, as such, have always existed in legal limbo and have episodically faced raids, seizures, shutdowns, and legal proceedings (Decorte, 2015; Hathaway & Rossiter, 2007). Jurisdictions that have legalized cannabis have approached community-based models in two ways. In Uruguay, for example, nonprofit self-managed cannabis clubs have been included in the cannabis legislation and are regulated by the government (Decorte et al., 2017). Membership is based on age and a monthly fee. People who use CTP are allowed to join. On-site consumption is permitted. In contrast, Canada's cannabis legislation (the *Cannabis Act*) was designed to create a government-sanctioned commercial market for recreational cannabis exclusively (Bennett & Young, 2022). Provinces and territories were tasked with enacting laws and regulations to enforce the *Cannabis Act* in their respective jurisdictions, resulting in more stringent enforcement by newly created enforcement units and concerted efforts to close existing community-based models (Capler & Bear, 2023). As noted by Capler and Bear (2023),

Legalization in Canada has had a stronger ability to dismantle and suppress the Compassion Club model than prohibition ever did. By removing the legal grey area, legalization either pushed Compassion Clubs fully inside a regulatory framework that excluded their core values, or it pushed them completely outside the zone of tolerance they had previously inhabited (p. 200).

In the province of British Columbia (BC), where we conducted our research, long-standing community-based models that had been operating since the 1990s (e.g., British Columbia Compassion Club Society^1^ and Vancouver Island Compassion Society^2^) were forced to close their doors following cannabis legalization because they faced government sanctions. Others, like the Blue Door dispensary, recently closed after being raided^3^ by BC's Community Safety Unit (CSU)—the unit responsible for compliance and enforcement of the *Cannabis Control and Licensing Act*. Sites that have remained open despite raids and administrative penalties, such as the Victoria Cannabis Buyers Club (VCBC), currently face an uphill battle to preserve the community-based model that many people who use CTP have relied on for decades. Our case study of VCBC was undertaken in this complex and rapidly changing policy environment. We identified VCBC as an important case study because it remained opened four years into cannabis legalization, continued to operate as a community access point for people who use CTP, *and* provided a consumption space to members (known as The Box).

Unlike other jurisdictions (Decorte et al., 2017), designated cannabis consumption spaces that allow indoor smoking are not permitted in Canada. Prior to legalization, concerns were raised by members of the Task Force on Cannabis Legalization and Regulation (Health Canada, 2016) about the disproportionate impact of smoke-free regulations that prohibit indoor smoking *and* limit outside smoking on structurally vulnerable populations. Others called for a harm reduction and equity-informed approach to cannabis consumption spaces (e.g., see Brown & Gagnon, 2018) highlighting that “unintended consequences of overregulation” such as eviction or consuming in spaces that pose more risks (e.g., cars) needed to be balanced with the goals of reducing exposure to second-hand smoke and discouraging smoking. It is worth noting that consumption spaces for people who smoke other psychoactive substances, such as stimulants, have been slow to implement in Canada compared to consumption spaces for people who inject, ingest, or snort (Gehring et al., 2022). The first legally sanctioned indoor safer inhalation site in North America opened in 2018 and closed in 2020 (Bourque et al., 2019; Gehring et al., 2022). To our knowledge, only one temporary legally sanctioned indoor safer inhalation site currently exists (e.g., The Harbour Inhalation Site in Victoria) and a handful of outdoor sites operate in BC.

The purpose of this paper is to present the findings of an instrumental case study of VCBC. Our main objective was two-fold. *Empirically*, we wanted to describe the experience of VCBC members and more specifically, members who smoke CTP and accessed The Box. This particular group of members is currently located at the intersection of three important issues arising from cannabis legalization in Canada. First, they use CTP. As noted above, people who use CTP in Canada have historically relied on community-based models to meet their needs. This has not changed following legalization (Ng et al., 2022). Second, they access cannabis via a middle-ground option (i.e., VCBC) that operates outside the government-run medical program and the government-sanctioned recreational market. Third, VCBC members smoke CTP, which poses a challenge because of smoking-free regulations and cannabis-specific regulations that came into effect following cannabis legalization (Gagnon et al., 2020; Sorensen et al., 2021). *Conceptually*, we wanted to explore ways of thinking more broadly about the experience of people who smoke CTP and the role of cannabis consumption spaces.

## The Case: VCBC

Founded in 1996, VCBC was modeled after the original San Francisco Cannabis Buyers Club. The San Francisco Cannabis Buyers Club was opened by Dennis Peron in 1991 to make medical cannabis available to people diagnosed with AIDS and people living with other chronic health conditions such as glaucoma, arthritis, and neurological disorders (Malott, 2009). By 1996, when VCBC opened, Peron and his small team were serving 12,000 members (Malott, 2009). VCBC started as a small grassroots project to provide compassionate access to cannabis to people living with chronic illnesses and symptoms (Smith & Kittel, 2019). In 2001, it secured the first location to provide members with a storefront experience and a consumption space. It has continued to do so despite having to move to another location in 2002 and more recently, in February 2023. In 2012, it became a registered nonprofit society governed by a board of directors (Smith & Kittel, 2019). Working with unregulated small-scale growers and collecting donations, VCBC was recording an average of $4,500 in daily sales in the year leading to cannabis legalization.

At the time of writing, VCBC staff served an average of 8,000 members in-person and online (J. Kittel, personal communication, March 30, 2023). Applying for a membership is free. However, each applicant is required to complete an application form, provide proof of condition and/or recommendation from a healthcare provider, and submit a copy of a photo ID. This means that all members have been screened for age requirements (19 years and older in BC) and health requirements. They have also agreed to a basic code of conduct (e.g., infection prevention measures, no scent policy, and tolerance zero for disruptive and disrespectful behaviors toward staff and other members). VCBC members have access to a comprehensive menu of affordable and high-quality cannabis and cannabis products. Cumulating more than two decades of experience in making cannabis products, the club offers products such as capsules, suppositories, topicals, and high-THC edibles. VCBC prices are more affordable than legal market prices. For example, a prerolled joint at VCBC is $3 below current BC market prices.^4^ VCBC dried flower ranges from $6 to $8 per gram, whereas market prices can go up to $13 per gram.^4^ High-THC (75 mg) cookies, which have proven particularly helpful for members with severe pain,^5^ sell for $2.50 each. In comparison, a cookie containing 10 mg of THC (the maximum dosage currently allowed under the *Cannabis Act*) costs $11.99^5^ in a licensed store.

The Box, VCBC's consumption space, was been accessible to members for more than two decades. In 2016, VCBC was granted an exemption^6^ from the municipal government under section 6(c) of its Cannabis-Related Business Regulation Bylaw to allow on-site consumption. As VCBC founder Ted Smith writes^7^:

The exemption granted to the VCBC is the first of its kind in the country but it is very clear every city in Canada should have spaces for patients to quietly consume their medicine protected from the weather, thugs and police harassment. As we march forward into a legal scheme, let us hope all levels of government work together to help manage these safe inhalation facilities.

**Figure 1.**
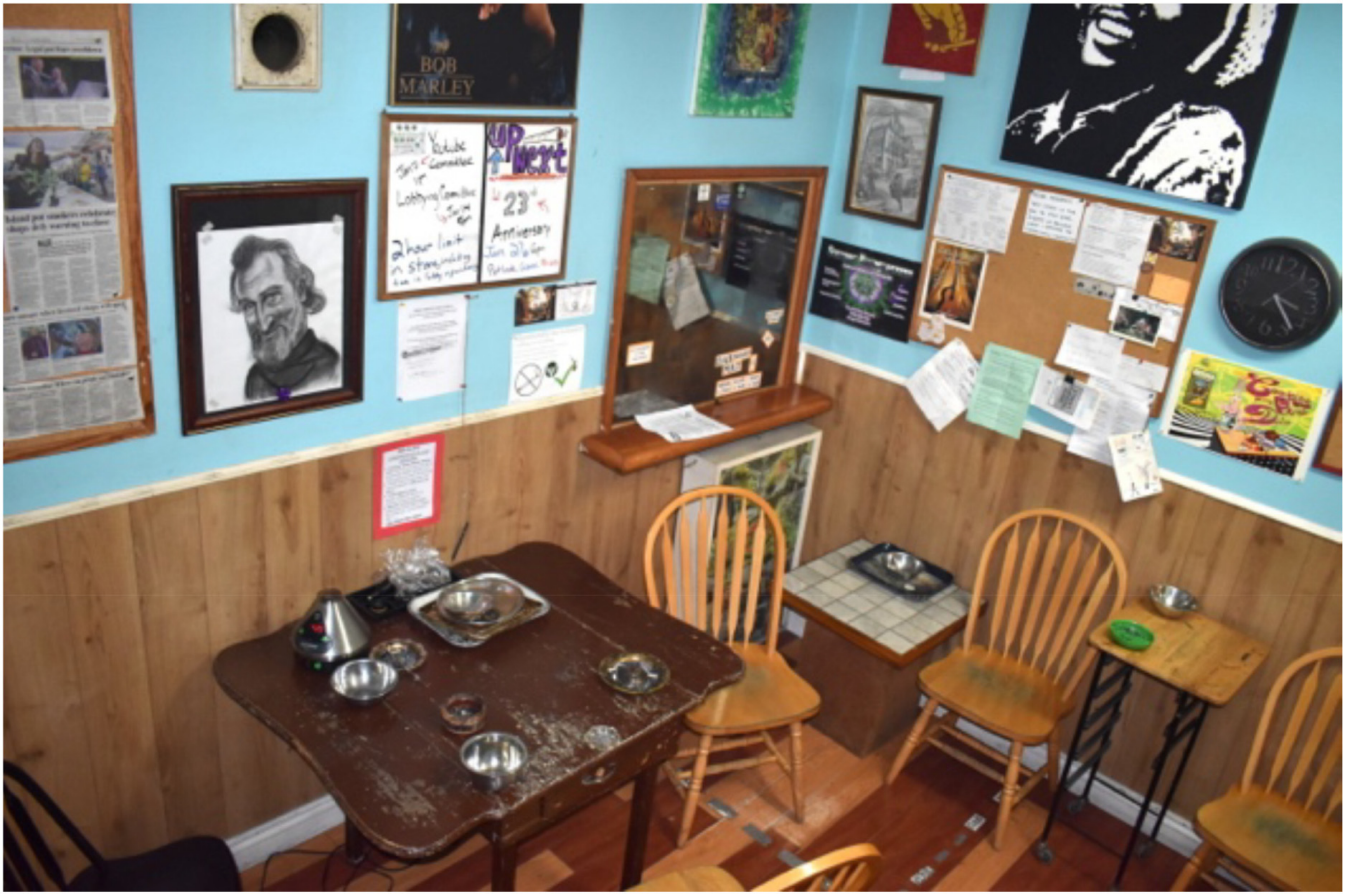
The Box (Smith & Kittel, 2019).

The Box (see Figure 1) included tables and chairs. Its walls were covered with art and bulletin boards. Equipment was available for members to prepare and consume cannabis on-site (e.g., trays, rolling paper, scissors, grinders, etc.). In December 2022, VCBC was served a notice of eviction^7^ after 22 years at the same location and relocated to a new location at the end of February 2023. Its commitment to providing “a dignified space [for members] to consume their medicine” (Smith & Kittel, 2019, p. 5) remained; however, The Box described above and in our findings no longer exists.

As we were conducting our case study, BC launched a round of public consultations to determine whether consumption spaces should be permitted (Ministry of Public Safety and Solicitor General, 2022a). The consultations were limited to recreational consumption and they excluded indoor smoking (and vaping). Nonetheless, survey respondents did indicate an interest in and support for indoor consumption spaces for people who smoke cannabis (Ministry of Public Safety and Solicitor General, 2022b). Among survey respondents who reported consuming cannabis, concerns about consumption spaces were notably lower than among those who did not consume cannabis (Ministry of Public Safety and Solicitor General, 2022b). Likewise, perceived benefits were higher among respondents who reported consuming cannabis than those who did not. Interestingly, a little over a third of the 16,092 respondents reported difficulties in accessing a space to smoke (or vape) because of provincial regulations, municipal bylaws, and strata and tenancy agreements (Ministry of Public Safety and Solicitor General, 2022b).

VCBC has a long history of legal victories for the rights of people who use CTP. Prior to legalization, it faced several raids by law enforcement and was successful in arguing that CTP is protected under the *Canadian Charter of Rights and Freedom.* In 2009, VCBC was involved in a court case that resulted in a landmark Supreme Court of Canada decision (*R. v. Smith*, 2015 SCC 34) after the person responsible for making its cannabis products was charged with possession of THC for the purpose of trafficking and possession of cannabis. In 2015, the Court ruled that prohibiting nondried forms of medical cannabis limited liberty and security of the person under section 7 of the *Charter* in a manner that is arbitrary and not justified under section 1, the provision allowing “reasonable limits” to be placed on *Charter* rights if certain conditions are met. This unanimous decision confirmed that people who use CTP have a constitutional right to consume cannabis in other forms (e.g., oils, teas, and baked goods). However, to this day, access to cannabis products that meet the needs of people living with chronic illnesses and symptoms remains challenging due to barriers inherent to the medical cannabis program, the THC limits set out in the *Cannabis Act*, costs, and access (mail delivery only).

After the *Smith*’s decision, VCBC continued to operate and maintained its services when the *Cannabis Act* came into force in October 2018. Following an inspection (May 2019) and two raids by the CSU (November 2019 and July 2020), VCBC was imposed a fine totaling $6.5 million for selling cannabis illegally (January 2022). A month later, VCBC was informed by the CSU that its website would be closed effective February 14, 2022. Our study was conducted during this uncertain period and amid the COVID-19 pandemic, which forced the temporary closure of The Box. The Box reopened in April 2022, one month after our survey ended. As we noted above, The Box in its original form closed on February 28, 2023, when VCBC moved to a new location. On March 23, 2023, the CSU conducted a third raid at the new location (Kittel, 2023) to seize VCBC's inventory and money. In response to this raid, VCBC announced it would be seeking a court injunction and taking legal action against the provincial and federal governments (Egan-Elliott, 2023).

### Study Design

Case study methodology as defined by Stake (1995, 2005) offers a flexible approach to exploring and describing a particular case in its real-life context. The case can be defined as an individual, a group, a program, a city, a country, or a particular phenomenon of interest (Stake, 1995, 2005). Stakes (1995, 2005) identifies three types of case studies: intrinsic, instrumental, and collective (or multiple). An intrinsic case study is undertaken to analyze the particularities of a unique case and develop a better understanding of this case alone (Stake, 1995, 2005). In contrast, an instrumental case study is primarily undertaken to examine a case that can provide insights into a broader phenomenon (Stake, 1995, 2005). When this approach is extended to multiple cases, it becomes a collective case study (or multiple case studies; Stake, 1995, 2005). Case study research starts from a simple yet complex question: “what can be learned from the single case?” (Stake, 2005, p. 443). For this study, we identified that VCBC and its consumption space could help us learn more about the experience of people who smoke CTP in a designated community space against a backdrop of cannabis legalization and the “slow eradication of compassionate access to cannabis.”^8^

Our case study was developed collaboratively with VCBC over the summer of 2021, during a period of great uncertainty for VCBC related to the pandemic and its legal standing. The study design was constrained by several contextual factors and informed by preparatory fieldwork at VCBC and consultation with the VCBC leadership team. First, it was understood that VCBC members were particularly vulnerable to COVID-19 and the safest way to collect data would have to be prioritized (Sevelius et al., 2020). Second, because The Box was temporarily closed, we had to find ways of reaching VCBC members when they came to VCBC in-person and when they received VCBC electronic communications. Third, the uncertain context and urgency of documenting the experience of members in the face of a possible permanent closure required flexibility and creativity. For all these reasons, we decided to conduct a descriptive survey that a convenience sample of VCBC members could complete online or using a hard copy. Case studies typically draw on multiple sources of data such as interviews, questionnaires, observations, documents, and so forth (Stakes, 1995, 2005). Like many other researchers conducting research during the COVID-19 pandemic (Krause et al., 2021; Sevelius et al., 2020; Shinew et al., 2022), we had to turn to other sources of data (e.g., news stories, VCBC documents, court documents, virtual discussion with VCBC leadership, and grey and scientific literature) to inform the survey development, contextualize the study and its findings, and open the analysis to conceptual inquiry.

Survey research provided opportunities to gather data in real-time during the COVID-19 pandemic, but it is not without limitations (Hlatshwako et al., 2021). In designing the study and survey, we used the following strategies to increase rigor and participation (Hlatshwako et al., 2021). We developed the survey with questions drawn from prior surveys of medical cannabis use (Walsh et al., 2013) and added questions related to The Box and its closure due to the COVID-19 pandemic. An interdisciplinary panel of experts from public health, psychology, nursing, social work, and policy reviewed the final draft of the survey. The leadership team at VBCB also provided input and ensured that the questions were written in a way that was clear and accessible to members. The final version of the survey had 34 questions covering four domains: demographics, cannabis consumption, access to and use of The Box, and the impact of a temporary closure of The Box due to COVID-19. We used an open-access survey platform and organized the questions to optimize flow. Providing a hard copy option was important to address the technology divide that became apparent during COVID-19 and thus, increase participation among VCBC members who are structurally vulnerable (e.g., precariously housed; Sevelius et al., 2020). Hard copies also provided a way for members with underlying health conditions and increased vulnerability to COVID-19 to pick up a survey at the same time as their cannabis products and then return it at their next visit—as opposed to being forced to fill out the survey on-site and spend more time in the shared space. Finally, we used existing electronic communication channels to recruit members for the survey who were using The Box before its temporary closure. This form of communication was important, especially for members who switched to online ordering during the pandemic.

### Data Collection and Analysis

After securing ethics approval, an invitation with an online link to the survey was sent to all VCBC members and also shared via VCBC's communications channels (e.g., social media and website until February 14, at which point the website was taken down by the CSU). Recruitment posters were displayed at VCBC, and paper copies of the survey were available to pick up or to fill out on-site. Completed hard copies of the survey were dropped into a locked box and picked up weekly by the lead researcher. Respondents were eligible to participate if they self-identified as VCBC members, used CTP, accessed The Box, and were able to complete the survey in English. Given the VCBC membership application process, which we describe above, we did not have to screen potential respondents for age requirements (19 years and older in BC) and health requirements. We recorded consent at the beginning survey and then asked respondents to answer mandatory questions to confirm eligibility. The rest of the survey questions were not mandatory. Respondents could opt to skip a question and continue with the survey. They also had the option of entering a draw for a $25 cash prize or a $25 VCBC gift card at the end of the survey.

The survey was open for responses between January and March 2022 and was completed by 104 respondents (*n *= 41 hard copies, *n *= 63 online). During this period, VCBC recorded approximately 2,700 in-person visits per month with the frequency of visits per person ranging from daily to weekly or monthly depending on where members are geographically located (J. Kittel, personal communication, April 4, 2023). It is worth noting that based on available geographical data, which provides an incomplete portrait of members, VCBC estimates that 1,214 members are based in Victoria and are most likely to access The Box (J. Kittel, personal communication, April 4, 2023). Based on this estimate, our sample (*n *= 104) represented 8.56% of this subgroup of members. Data collected from the hard copies were entered into the online survey platform by a research assistant. A survey was deemed completed if mandatory questions confirming eligibility were answered affirmatively, and the participant proceeded to answer the questions until the end of the survey, at which point it was submitted. The surveys of respondents who skipped some of the nonmandatory questions were not considered incomplete.

Descriptive statistics were generated from the survey data (Thrane, 2022). For Likert scale scores, we opted to use the weighted averages available via the survey platform, meaning that the highest the score on the Likert scale the more weight it was given in the calculation of the average (i.e., each score on the scale did not carry the same weight; Lavallée & Beaumont, 2015). Using the Likert scale findings and the sources of data collected as part of the case study, which we detailed above, we also identified three conceptual avenues that can inform future research and policy-making. This is consistent with our overall objective for this case study which was to think more broadly about the experience of people who smoke CTP and the role of cannabis consumption spaces.

## Results

The results section is divided into two parts. The first part describes the sociodemographic and socioeconomic characteristics of respondents, followed by their cannabis consumption and overall experience accessing The Box. The second part identifies three conceptual avenues that are then carried over into the discussion.

### Descriptive Results

#### Sociodemographic and Socioeconomic Characteristics

58% (*n* = 60) of respondents were 40 years and older, and 80% identified as cisgender men or women (*n* = 83; Table 1). While the majority (70%, *n* = 60) identified as White (European descent), it is worth noting that 13% (*n* = 13) identified as Indigenous (First Nations, Métis, Inuit). Of particular importance to this study, 69% (*n* = 72) of the respondents were living in a multidwelling unit where smoke-free regulations apply. Only 2 (2%) of those respondents were property owners (e.g., condos). The remaining were renters or residents (e.g., assisted living, temporary housing, etc.). Of those living in a house (19%, *n* = 20), a third were renters, which means that smoke-free regulations most likely applied as well. In total, 6% of respondents were unhoused or temporarily housed.

**Table 1.** Sociodemographic and Socioeconomic Characteristics.

	*n* (%)
Age (years)	
19–20	2 (1.94)
21–30	14 (13.59)
31–40	27 (26.21)
41–50	18 (17.48)
51–60	18 (17.48)
61–70	16 (15.53)
>71	8 (7.77)
Gender	
Cisgender man	52 (50)
Cisgender woman	31 (29.8)
Transgender man or women, nonbinary, gender queer, two-spirit^a^	12 (11.52)
Prefer not to answer	9 (8.65)
Ethnicity^b^	
Indigenous	13 (12.5)
Arabic descent	3 (2.88)
East, South, or Southeast Asian descent	8 (7.69)
West Asian or Middle Eastern descent	1 (0.96)
African or Caribbean descent	4 (3.85)
Latin American descent	1 (0.96)
European descent (White)	73 (70.1)
European descent (non-White)	4 (3.85)
Prefer not to answer	4 (3.85)
Other	3 (2.88)
Housing	
Unhoused	3 (2.88)
Shelter or temporary housing	5 (4.81)
Assisted living	9 (8.65)
Renting an apartment	43 (41.35)
Renting condo or strata	6 (5.77)
Renting house	6 (5.77)
Owning a condo or strata	2 (1.92)
Owning a house	14 (13.46)
Renting a room	8 (7.69)
Subsidized housing	4 (3.84)
Other	4 (3.84)
Income	
<$20,000	44 (44)
$20,000–$39,999	29 (29)
$40,000–$59,999	12 (12)
>$60,000	15 (15)
Sources of income^b^	
Social assistance	8 (7.77)
Disability assistance	49 (47.57)
Full-time employment	22 (21.36)
Part-time employment	16 (15.53)
Contract-based employment	8 (7.77)
Personal business	6 (5.83)
Gig work	8 (7.76)
Work “under the table”	9 (8.74)
Self-employment	13 (12.62)
Pension/employment insurance	14 (13.59)
Other	4 (3.88)

*Note.* Some columns may not add up to 100% as participants were able to skip questions.

^a^ Gender categories were aggregated to protect the confidentiality of respondents.

^b^ Select all that apply questions.

**Figure 2.**
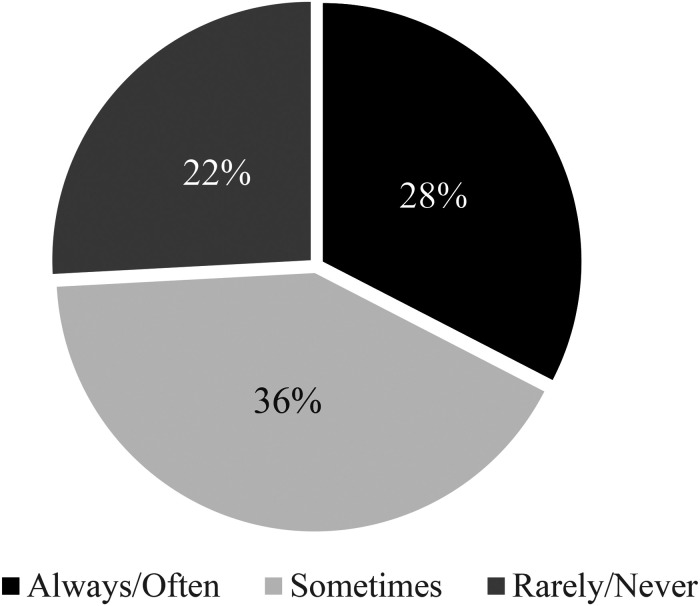
Affordability of cannabis.

When asked about their annual income, 44% (*n* = 44) reported an income of <$20,000, 41% (*n* = 41) between $20,000 and $39,999, 12% (*n* = 12) between $40,000 and $59,999, and 15% (*n* = 15) $60,000 and more. Sources of income varied across the sample, but it is worth highlighting that close to 50% of the sample identified government disability assistance as a main source of income. The average amount respondents spent on cannabis per month was $368.20. 61.1% (*n *= 63) reported that they can “always” or “often” afford their cannabis each month. However, 28% (*n *= 29) stated that they “always” or “often” had to choose between paying for cannabis and other necessities such as rent or food. For 36% (*n* = 37) of respondents, this dilemma was less frequent (i.e., “sometimes”), but it was nonetheless something they experienced (see Figure 2).

#### Cannabis Consumption

Table 2 summarizes the information collected about respondents’ cannabis consumption including frequency, reason(s), preferred modes of consumption, and amount used. Frequency was reported according to monthly, weekly, and daily consumption. 73% (*n* = 75) of respondents were daily users. Of the daily users, 45% (*n* = 46) reported consuming cannabis more than four times per day, and 28% (*n *= 29) between one to four times a day. The majority of respondents who identified as weekly users reported consuming cannabis three to six times a week (16%, *n* = 16). Only 5% (*n *= 5) of the sample reported monthly cannabis consumption. When asked to select one or more reasons for consuming cannabis, 80% (*n* = 83) of respondents selected therapeutic use for an illness or chronic condition, and 77% (*n* = 80) stated that they used cannabis more specifically for physical and mental wellness. Other reasons included recreational or social use (48%, *n* = 50) and spirituality or traditional healing (34%, *n* = 35). We will revisit these answers in the next section.

**Table 2.** Cannabis Consumption.

	*n* (%)
Frequency	
1–3 times per month	5 (4.85)
1–2 times per week	7 (6.79)
3–6 times per week	16 (15.5)
1–2 times per day	11 (10.67)
3–4 times per day	18 (17.47)
> 4 times per day	46 (44.66)
Reason(s)^a^	
Therapeutic use for an illness or chronic condition	83 (79.81)
For physical and mental wellness	80 (76.92)
For recreational or social purposes	50 (48.08)
For spiritual or traditional healing purposes	35 (33.65)
Other	5 (4.80)
Preferred mode of consumption^a^	
Eating	55 (52.88)
Drinking	17 (16.35)
Joints with tobacco	14 (13.46)
Joints without tobacco	65 (62.50)
Pipe	44 (42.30)
Water pipe or bong	29 (27.88)
Vaporizing and vaping	35 (33.65)
Applying it on the skin	30 (28.85)
Dabbing	35 (33.65)
Bath bomb	2 (1.92)
Suppositories	3 (2.88)
Other	4 (3.84)

*Note.* Some columns may not add up to 100% as participants were able to skip questions.

^a^ Select all the apply questions.

The most to the least preferred mode of consumption included: smoking, ingesting (eating or drinking), and topical application (lotions or bath bombs). Of the modes of smoking, the three most commonly reported were smoking joints without tobacco (63%, *n *= 65), using a pipe (42%, *n = *44), and vaporizing, vaping, and dabbing (34%, *n* = 35)*.* Given that participants used various modes of consumption, we asked respondents to estimate the amount of cannabis they consumed daily in a text box using the metric of their choice (e.g., grams of flower, number of joints, etc.). The majority of respondents provided quantities in grams (daily range from 0.25 to 7 g) or in the number of joints smoked (daily range from 0.5 to 6 joints). Some respondents stated it was difficult for them to estimate the quantity of cannabis consumed daily or that their daily consumption fluctuated based on their symptoms that day or their ability to purchase cannabis. Others did not report quantity but rather a concentration in mg (e.g., mg of THC), which did not allow us to calculate a daily amount per se. Finally, respondents who consumed edibles in addition to smoking provided quantities of the edible (e.g., two squares of a chocolate bar included on VCBC's menu).

#### Experience Accessing the Box

We asked respondents how often they accessed The Box before its temporary closure, what equipment they used (if any), how much they smoked during a regular visit, why they smoked in The Box, and how beneficial the space had been to them (see Table 3). In terms of frequency of visits, the sample was split into three groups: (a) monthly visitors (31%, *n* = 30) ranging from one to three visits per month, (b) weekly visitors (47%, *n* = 45), most of whom accessed The Box two to four times a week, and (c) daily visitors (22%, *n *= 21) accessing The Box one to two times a day. The equipment most commonly used by respondents included grinders (55%, *n *= 48), dab rigs (42%, *n *= 37), and the Volcano Vaporizer (33%, *n *= 29). Under “other,” respondents mentioned rolling trays, rolling paper, ashtrays, and alcohol swabs. The majority of respondents (86%, *n *= 76) reported smoking an average of 1 to 2 joints per visit.

**Table 3.** Experience Accessing The Box.

	*n* (%)
Frequency of use	
Once a month	14 (14.58)
2–3 times a month	16 (16.67)
Once a week	6 (6.25)
Twice a week	16 (16.67)
3–4 times a week	17 (17.71)
5–6 times a week	6 (6.25)
Once a day	9 (9.38)
Twice a day	11 (11.46)
3–4 times a day	1 (1.04)
Equipment used^a^	
Dab rig	37 (42.05)
Volcano vaporizer	29 (32.95)
Grinder	48 (54.55)
Scissors	27 (30.68)
Rolling paper	13 (14.77)
Other	11 (12.5)
Reason(s) for smoking in The Box^a^	
I enjoy socializing with others	68 (69.38)
I feel safer smoking in The Box	50 (51)
I can learn from others	49 (50)
I like being around others when they consume cannabis	42 (42.86)
I do not want to smoke outside	40 (40.82)
I can help others	37 (37.76)
I can share cannabis with others	35 (35.71)
I cannot smoke where I live because smoking is banned	31 (31.63)
I can dab high-THC concentrates	26 (26.53)
I am concerned about getting a ticket	17 (17.35)
I am concerned about being evicted	17 (17.35)
I cannot smoke where I live because of roommates, relatives, or children	14 (14.29)
Other: Please specify	14 (14.28)
Reasons why The Box has been beneficial^a^	
I made new friends and connections	66 (67.34)
I learned new information about cannabis	63 (64.29)
I learned new information about cannabis products	62 (63.27)
I felt a sense of belonging and community	62 (63.27)
I became less isolated	61 (62.24)
I looked forward to visiting The Box	59 (60.20)
I shared with someone who could not afford their medicine that day	45 (45.92)
I learned about existing community services and activities	45 (45.92)
I adjusted my cannabis use because of information I learned in The Box	40 (40.82)
I used equipment I would not otherwise have access to	39 (39.80)
I was shown how to safely consume cannabis	36 (36.73)
I received medicine that a member shared with me	31 (31.63)
I used the volcano vaporizer provided instead of smoking	30 (30.61)
I was shown how to effectively prepare cannabis for consumption	27 (27.55)
I got help to prepare my cannabis for consumption	26 (26.53)
Other: Please specify	10 (10.20)

*Note.* Some columns may not add up to 100% as participants were able to skip questions.

^a^ Select all the apply questions.

Socializing was a common reason (69%, *n* = 68) for accessing The Box, followed by feeling safer smoking in The Box (51%, *n* = 50), learning from others (50% *n* = 49), enjoying being around others^9^ when consuming cannabis (43%, *n* = 42), helping others (36%, *n* = 35), sharing cannabis with others (36%, *n* = 35), and accessing other smoking modalities such as dabbing high-THC concentrate (27%, *n* = 26). Needing a place to smoke was also an important reason to access The Box. This was true for respondents who did not want to smoke outside (41%, *n* = 40) and those who could not smoke at home because of second-hand smoke (14%, *n* = 14) and/or smoking bans (32%, *n* = 31). Of particular importance to this study, close to 20% of respondents reported accessing The Box because they were concerned about being evicted for smoking indoors or ticketed for smoking outdoors.

The main benefits of The Box were: (a) breaking isolation and building community, (b) learning something new and adjusting cannabis use, and (c) reducing potential harms associated with cannabis. Respondents reported making new friends and connections (67%, *n *= 66), gaining a sense of belonging and community (63%, *n* = 62), becoming less isolated (62%, *n *= 61), learning about community services and activities (46%, *n *= 45), sharing with someone who could not afford cannabis that day (46%, *n *= 45) or being offered cannabis by someone who wanted to share (32%, *n *= 31), and getting help from someone to prepare cannabis (27%, *n *= 26). They also learned something new while using The Box including new information about cannabis (64%, *n *= 63), cannabis products (63%, *n *= 62), equipment (40%, *n *= 39), and cannabis preparation (28%, *n *= 27). Interestingly, 41% (*n* = 40) had adjusted their cannabis use based on what they had learned in The Box. Along the same lines, 37% (*n* = 36) had been shown how to use cannabis safely, and a third of respondents had switched their smoking modality from smoking to vaporizing. Under “other” respondents reiterated the role of The Box in breaking isolation and building community. “The Box is like a village,” one respondent wrote.

### Conceptual Avenues

#### Cannabis is Therapeutic, and so is The Box

**Figure 3.**
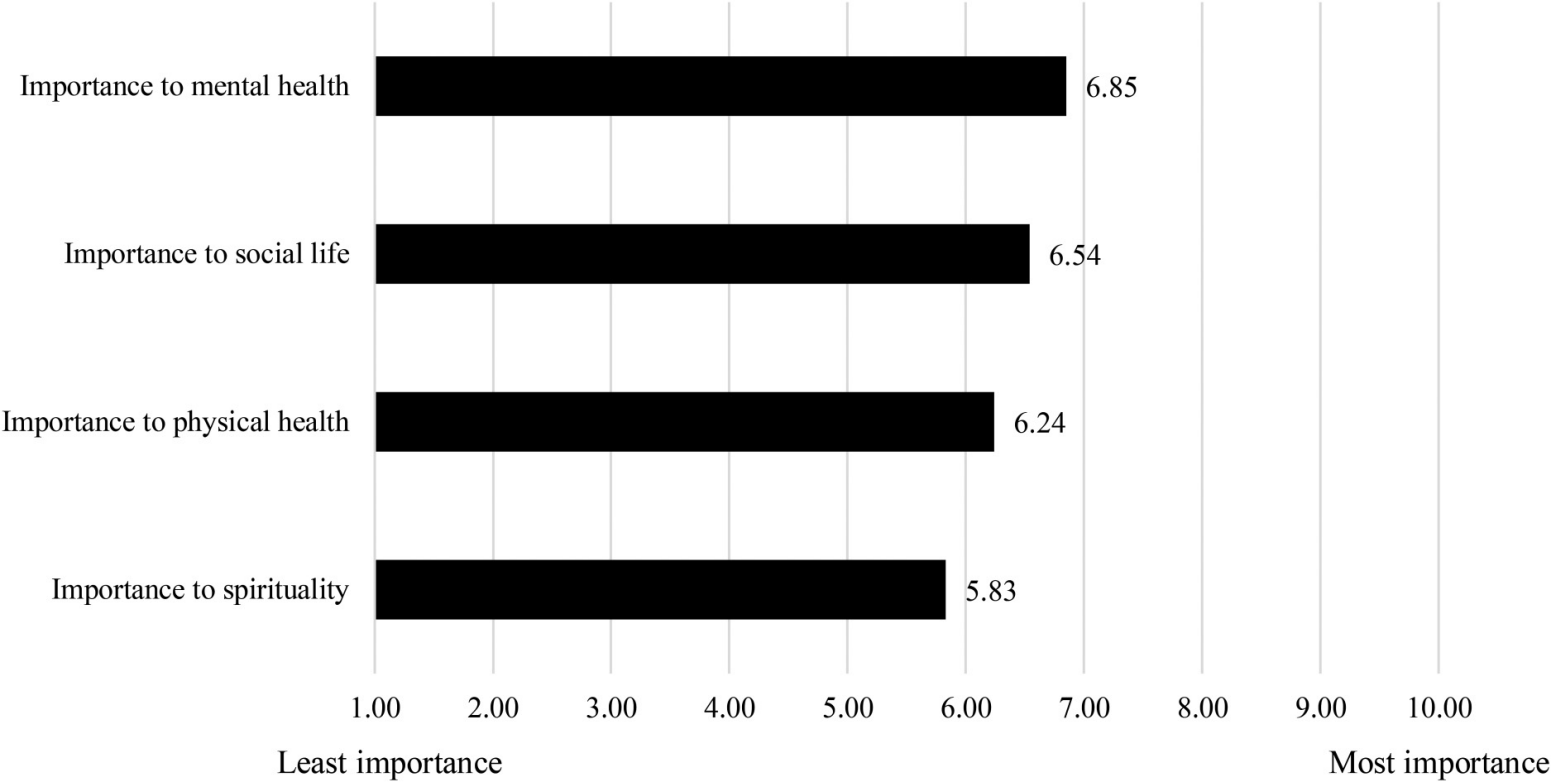
Importance of The Box.

As pointed out above, the reasons why respondents consumed cannabis varied and were not exclusively therapeutic in a medical sense (i.e., symptom relief). Managing an illness or chronic condition was common but so was physical and mental wellness. Recreational or social use, which privileges enjoyment and pleasure, was also deemed important. Finally, for a third of our sample, cannabis was used for spiritual or traditional healing purposes as well. The Box offered a place where cannabis could be consumed with all these reasons in mind. For example, when we asked respondents to use a Likert scale of 1 to 10 (10 being the most important and 1 being the least important) to rank the importance of The Box on their mental health, physical health, social life, and spirituality (Figure 3), they reported the following: Mental health was the single most important contribution of The Box with a weighted average of 6.85. Social life was ranked second at 6.54. Importance to physical health was ranked third with a weighted average of 6.24, followed by spirituality with a weighted average of 5.83.

Considering that smoking was the preferred mode of consumption among our respondents, it makes sense that accessing a place to smoke would be deemed important, especially when options to smoke indoors and outdoors might be limited. However, our findings suggest that The Box was more than “just a place to smoke” for respondents. For example, mental health and social life ranking higher than physical health are indicative of space-specific effects that merit further inquiry. In other words, if the sole purpose of a cannabis consumption space is to smoke cannabis (as a medicine), other dimensions of the consumption experience *in that space* would likely not come through in the survey. As such, a potential conceptual avenue to explore is why and how having access to a space like The Box generates broader therapeutic effects beyond the therapeutic effects of cannabis per se. This could also help broaden the concept of CTP to include other dimensions of health and well-being, which are well represented in the survey findings.

#### If not in The Box, then Where

**Figure 4.**
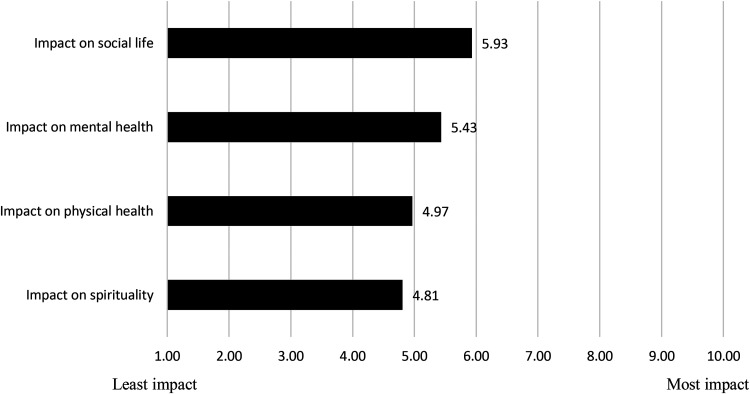
Impact of closure.

When we asked about the impact of the temporary closure of The Box due to COVID-19 using the same Likert scale design (Figure 4), one respondent used the text box and described it as follows: “It was a loss. A social, medicinal loss.” Consistent with the above findings, the *social loss* was deemed the most important, followed by the *medicinal loss*. In total, 67% (*n *= 63) of our sample stated that they had lost contact with other members and friends and 62% (*n *= 58) reported feeling more isolated. About a third of respondents also reported a lost connection with the VCBC (32%, *n *= 31). Precisely, 23% (*n *= 22) experienced a worsening of their mental health due to the closure, while 12% (*n *= 11) experienced a worsening of their physical health. Because of the closure, 17% (*n *= 16) reported smoking less, and 7% (*n *= 7) switched to another mode of delivery (i.e., edibles). Some noted that the closure had made it harder for them to manage symptoms (14%, *n *= 13). Under “other,” respondents mentioned their inability to access an alternative safe space to smoke cannabis or smoke outside, worsening symptoms for which they use CTP, and lacking access to equipment.

Given that smoking in The Box generated benefits that exceeded those associated with smoking cannabis (as a medicine), that smoking was the primary mode of consumption for respondents, and that smoking posed real challenges (e.g., smoking bans) and risks (e.g., eviction), we see two potential conceptual avenues to explore. First, it is important to untangle the broad concept of “people who use CTP.” Studying those who primarily or exclusively *smoke* CTP as a distinct group would generate a more nuanced understanding of reasons why they smoke, where they can or cannot smoke (and how this impacts them), what role consumption spaces play in their ability to self-medicate but also to experience additional benefits (e.g., mental health, social, spiritual, etc.). This brings us to the second conceptual avenue, the need to rethink consumption spaces for people who smoke CTP as having the potential to generate benefits. Our survey points to individual health and public health benefits that are worth exploring. Conceptually merging all forms of indoor smoking as equally harmful runs the risk of overlooking such benefits.

#### Feeling (Un)Safe in The Box

We asked respondents to rank how safe they felt in The Box on a Likert scale of 1 to 10 (10 being the safest and 1 being the least safe). The responses yielded a weighted average of 8.75, with 82% (*n* = 80) of the sample ranking safety between 8 and 10. When asked if they ever felt unsafe in The Box, 44 respondents entered a comment. Fifteen comments detailed two main situations that made them feel unsafe: overcrowding and ventilation in The Box as well as aggressive behaviors and inappropriate comments (e.g., racist and sexist remarks). Rapid intervention from the staff in such situations was noted by respondents who commented about aggressive and inappropriate comments in The Box. We noted two important areas that need further conceptual analysis.

First, we see a need to dive more deeply into the concept of safety. For example, The Box may feel like a “refuge haven” (an expression used by a respondent) because it offers protection from the risks of smoking outdoors (e.g., ticketing) or in indoor spaces where smoking is prohibited (e.g., eviction). However, the same feeling of safety may not apply *inside* The Box. How we acknowledge these tensions are unclear and it warrants more work to ensure that in the search for safety, VCBC members are not forced into an unsafe space. We see potential opportunities for joint work with scholars who are studying other types of consumption spaces such as supervised injection/inhalation sites or drug consumption rooms. Second, more work is needed to conceptualize safety in the design of cannabis consumption spaces to address concerns related to overcrowding and ventilation. Research conducted on the first regulated safer inhalation site in North America (opened in Lethbridge, Alberta, in 2018 and subsequently closed in 2020) could be helpful because it outlines many design considerations that have not traditionally been included in cannabis consumption spaces literature (Bourque et al., 2019). Furthermore, it provides important evidence on how to design an inhalation space that protects both staff and users.

## Discussion

Our findings offer important insights into a unique community-based model of cannabis cultivation, distribution, and consumption and a unique group of people who use CTP, both of which are currently located outside the scope of the *Cannabis Act*. Much like the original cannabis buyers club on which it was modeled and other cannabis clubs alike, VCBC ensures safe, low-barrier access to affordable, high-quality products for people who use cannabis as a medicine. It also serves the complementary function of providing a space for social interaction and community building—which may play a particularly important therapeutic role in the context of this still stigmatized and marginalized medicine. VCBC also provides a concrete solution to the three policy problems described above, all of which remain firmly in place despite cannabis legalization.

First, it addresses a major gap by providing a middle-ground option for people who face numerous structural vulnerabilities and live with chronic illnesses/disabilities whose needs are not met by the government-sanctioned market—a market intended for *recreational* cannabis consumption. It also provides members, most of whom are renters or precariously housed, access to a space where they can smoke. Second, it provides a low-threshold pathway for people who want to (e.g., self-medicating) or have to (e.g., no access to or support from a physician) use CTP outside the medical system—a system that continues to present many limitations because, as Ng et al. (2022) point out, “pathways for access and use of medical cannabis [in Canada] have largely remained the same” (p. 4) since cannabis was legalized. Finally, it provides storefront access to cannabis products for people who use CTP as well as access to education and support. Under the *Cannabis Act*, storefront access is limited to people who use cannabis for recreational purposes and services such as providing advice for managing health conditions or symptoms are not permitted.

Surveying VCBC members allowed us to identify who uses The Box and why they do so. The majority of our respondents identified as cisgendered White men and reported a yearly income of <$40,000. This is consistent with other Canadian studies conducted with people who use CTP (Belle-Isle et al., 2014; Capler et al., 2017; Costiniuk et al., 2019; Lucas 2012; Walsh et al., 2013). Across these studies, 67%–87% of participants identified as cisgendered men, 81%–93% identified as White, and 48%–89% reported a yearly income of <$40,000. In contrast to previous studies, however, our survey generated higher engagement from people who identified as transgendered, nonbinary, genderqueer, and/or Two-Spirit as well as people who identified as Indigenous. While our study design did not include an in-depth exploration of Lesbian, Gay, Bisexual, Transgender, Queer/Questioning, Intersex, Asexual, Two-Spirit (LGTBQIA2S) + and Black, Indigenous, and People of Color (BIPOC) voices and experiences, we see a need for more research to address this gap.

Respondents used The Box because smoking was their preferred (and most effective) mode of delivery and having a place to smoke was important not only to optimize symptom management but also to build meaningful social connections and feel better mentally while seeking refuge from the potential risks of smoking outside (e.g., ticketing) or in places where smoking bans are in effect (e.g., eviction). This is a significant finding for two reasons. One, it is consistent with research dating as far back as the 1990s which found that cannabis clubs worked at the medical–social–mental–spiritual nexus (Feldman & Mandel, 1998; Hathaway & Rossiter, 2007). In other words, the impact of cannabis clubs extends beyond cannabis itself. Second, it makes an original contribution to the existing literature by revealing particular aspects of cannabis legalization that have not been studied to date, namely by conducting a case study of a cannabis club that continues to operate outside the legal framework and exploring the role/importance/contribution of consumption spaces in a context where smoking indoor *and* outdoor has become more difficult and riskier for people who use CTP.

When asked about the importance and helpfulness of The Box, more specifically, our respondents reported reasons and benefits that are best interpreted by combining public health, harm reduction, and wellness perspectives. Taken together, these perspectives help us understand why consumption spaces matter to people who *smoke* CTP, and more importantly, it moves the analysis beyond the narrow public health focus of second-hand smoke, which continues to drive policy-making at all levels of government in Canada (Fischer et al., 2020). Based on our findings and the existing literature on community-based models of cannabis cultivation, distribution, and consumption, we see several considerations that should be incorporated into policy-making to engage in a balanced and equity-informed analysis of consumption spaces and cannabis clubs more broadly. Echoing the literature summarized above, we conclude by discussing what we see as the public health, harm reduction, and wellness benefits of allowing indoor consumption spaces for people who use CTP. From a *public health perspective*, our findings suggest that The Box contributes to reducing exposure to second-hand smoke for nonsmokers by allowing people who need to smoke to gather in one designated space. Furthermore, access to this space is contingent upon a membership system, which includes a verification process (e.g., age and medical condition requirements) that seeks to limit the use of The Box to therapeutic users and adults. The quality of and information about the cannabis products smoked in that space can also contribute to reducing public health risks associated with cannabis use, such as intoxication, contamination, and problematic use. And as our findings indicate, The Box increases access to education, guidance, and equipment resulting in positive changes in behaviors. Overall, this is consistent with the purpose of the *Cannabis Act*^10^ and previous studies (Belackova et al., 2016; Pardal et al., 2022; Parés-Franquero et al., 2019)

From a *harm reduction* perspective, The Box can actively reduce potential harms associated with cannabis by allowing members to exchange information and advice, help each other, access equipment, and use more in less harmful ways in the presence of others. By allowing members to smoke in a designated area, it also reduces potential harms associated with not having a place to smoke, such as poorly managed symptoms and use of unregulated/unknown quality cannabis products that may be more difficult to dose and manage alone (e.g., edibles and oils), both of which were reported in our findings. Our findings also indicate that The Box plays a role in reducing potential social (e.g., eviction) and policy (e.g., ticketing) risks associated with cannabis smoking, which, in turn, generates a sense of safety. As Obradors-Pineda et al. (2021) point out in a recent paper on the harm reduction potential of cannabis clubs, “social and legal risks of cannabis use are substantially reduced in a club setting, which also provides a safe environment for consumption along with information on normative amounts of cannabis that can be used daily and monthly” (p. 3). This is consistent with our findings. It is also consistent with research by Belackova et al. (2016) on the harm reduction benefits of cannabis clubs in Spain involving focus groups with close to 100 club representatives across all five federations, each serving on average 100 to 250 members.

Finally, from a *wellness* perspective, our survey confirms that the social and mental (and to some extent spiritual) impact of The Box is considerable. This echoes earlier work on cannabis clubs as well as more recent efforts to adopt a wellness framework to understand why and how people use cannabis. Subrizky (2018) explains that public health and harm reduction perspectives focus on problems, risks, and harms associated with cannabis use to the detriment of pleasure, social connections, relaxation, and so forth. Our findings are consistent with what DeAngelo (2015), founder of Harborside Health Centre, points out in The Cannabis Manifesto: “Neither medical nor recreational fully nor accurately described the way I saw people use cannabis. I suspected that there was a third category but didn't know how to analyze or describe it” (p. 66). Wellness, he argues, is the missing perspective that can help us understand the overlapping reasons why people use cannabis and what role consumption spaces play in well-being, not simply symptom management. More research is needed to move past the recreational and medical divide when studying the experience of people who use cannabis to feel better physically, mentally, socially, and spiritually. We hope that our findings can provide some potential avenues for this work, given that wellness is well represented in the data.

Our survey had some limitations, some of which are discussed in the study design section. The scope of the study was narrow, the context was in flux amid a pandemic and ongoing enforcement measures (e.g., website closure, risk of raids, etc.), we relied on a survey as a primary data collection method, the survey questions focused primarily on the experience of using The Box, and the sample was a small proportion (8.56%) of a subgroup of VCBC members. In opting for a convenience sample of members with a common experience of (and need for) using The Box, this may have generated a positive bias. However, critiques offered by respondents reflected an ability to recognize the benefits of The Box while also acknowledging its limits. Furthermore, it is important to recognize that members for whom The Box was the only consumption space available may have responded favorably in the hopes of preserving that space in a time of heightened uncertainty. Finally, we cannot claim that our sample was representative of members nor that our findings are generalizable to all VCBC members—or people who use CTP more broadly. As noted above, our sample was consistent with other Canadian studies conducted with people who use CTP; thus suggesting potential transferability.

## Conclusion

Our study adds to the literature on cannabis clubs and raises important issues related to cannabis legalization, the role of community-based models of cultivation, distribution, and consumption, and the impact of accessing (and potentially losing access to) a consumption space such as The Box. Overall, we consider that VCBC and its on-site consumption space offered a useful case study to make “real-time observations” (Watson et al., 2019) about cannabis legalization as it unfolds over time and across jurisdictions. Given that the BC government, which is tasked with enforcing the *Cannabis Act* and cannabis-related provincial statutes and regulations, has been closing community-based models of cannabis cultivation, distribution, and consumption, the timing of our findings is important. VCBC is currently the last remaining community-based model in the province, and it faces uphill administrative and legal battles. More equity-informed research is needed to document the experiences of people who access CTP outside the government-run medical program and the government-sanctioned recreational market. With the closure of community-based models cultivation, distribution, and consumption in BC, many have seen their access to CTP decrease and have lost access to community, education, and support, and a storefront experience. An equity-informed approach to policy-making is also needed to understand how laws and regulations, such as laws and regulations prohibiting indoor cannabis consumption spaces, impact people who experience intersecting structural vulnerabilities (e.g., poverty, disability, criminalization, etc.). Our survey serves as a reminder that for people who smoke CTP, accessing a community-based indoor consumption space can be therapeutic. If consumption spaces are to be introduced, as the consultations conducted by the BC government seem to suggest, granting broader community access to these spaces and allowing for safer indoor smoking merit close consideration.

## Notes

1. See notice of closure (British Columbia Compassion Club Society, 2022).

2. See Crescenzi (2019).

3. See Milloy and Gagnon (2022).

4. Calculated based on summer 2022 listed prices on https://www.bccannabisstores.com/.

5. See Rudisuela (2021) and Wyton (2022).

6. See Smith (2016).

7. See *Victoria News Staff* (2023).

8. Quote by lawyer Jack Lloyd in Hager and Imam (2020).

9. It is important to clarify that “enjoying being around others” is different from socializing and includes reasons such as the shared experience of smoking cannabis with others, the therapeutic benefits of inhaling the smoke of others, and the cost-saving aspect of medicating with the second-hand smoke of others.

10. The purpose of the *Cannabis Act* is to protect public health and public safety and, in particular, to (a) protect the health of young persons by restricting their access to cannabis; (b) protect young persons and others from inducements to use cannabis; (c) provide for the licit production of cannabis to reduce illicit activities in relation to cannabis; (d) deter illicit activities in relation to cannabis through appropriate sanctions and enforcement measures; (e) reduce the burden on the criminal justice system in relation to cannabis; (f) provide access to a quality-controlled supply of cannabis; and (g) enhance public awareness of the health risks associated with cannabis use.

## References

[bibr3-00914509231183147] Belackova V. Tomkova A. Zabransky T. (2016). Qualitative research in Spanish cannabis social clubs: “The moment you enter the door, you are minimizing the risks”. International Journal of Drug Policy, 34, 49–57. 10.1016/j.drugpo.2016.04.009 27461986

[bibr4-00914509231183147] Belackova V. Wilkins C. (2018). Consumer agency in cannabis supply – exploring auto-regulatory documents of the cannabis social clubs in Spain. International Journal of Drug Policy, 54, 26–34. 10.1016/j.drugpo.2017.12.018 29367012

[bibr5-00914509231183147] Belle-Isle L. Walsh Z. Callaway R. Lucas P. Capler R. Kay R. Holtzamn S. (2014). Barriers to access for Canadians who use cannabis for therapeutic purposes. International Journal of Drug Policy, 25(4), 691–699. 10.1016/j.drugpo.2014.02.009 24947993

[bibr6-00914509231183147] Bennett R. Young A. (2022). Canada’s Cannabis Act: Annotation & commentary. LexisNexis.

[bibr7-00914509231183147] Bourque S. Pijl E. M. Mason E. Manning J. Motz T. (2019). Supervised inhalation is an important part of supervised consumption services. Canadian Journal of Public Health, 110, 201–215. 10.17269/s41997-019-00180-w PMC6964381 30725386

[bibr9-00914509231183147] British Columbia Compassion Club Society (2022). 2 month notice of closure. https://thecompassionclub.org/2-month-notice-of-closure/

[bibr10-00914509231183147] Brown M. Gagnon M. (2018). Why we need ‘safe’ spaces to consume cannabis. https://ottawacitizen.com/opinion/columnists/brown-and-gagnon-why-we-need-safe-spaces-to-consume-cannabis

[bibr11-00914509231183147] Capler R. Bear D. (2023). From compassion to commercial: What got left behind in the transition to legal cannabis in Canada. In Pardal M. (Ed.), The cannabis social club (pp. 155–186). Routledge.

[bibr12-00914509231183147] Capler R. Walsh Z. Crosby K. Belle-Isle L. Holtzman S. Lucas P. Callaway R. (2017). Are dispensaries indispensable? Patient experiences of access to cannabis from medical cannabis dispensaries. International Journal of Drug Policy, 47, 1–8. 10.1016/j.drugpo.2017.05.046 28667878

[bibr13-00914509231183147] Chayama K. L. Valleriani J. Ng C. Haines-Saah R. Capler R. Milloy M.-J. Small W. McNeil R. (2021). The role of cannabis in pain management among people living with HIV who use drugs: A qualitative study. Drug and Alcohol Review, 40(7), 1325–1333. 10.1111/dar.13294 33843074 PMC8580359

[bibr14-00914509231183147] Costiniuk C. T. Saneei Z. Salahuddin S. Cox J. Routy J. P. Rueda S. Abdallah S. J. Jensen D. Lebouché B. Brouillette M. J. Klein M. Szabo J. Frenette C. Giannakis A. Jenabian M. A. (2019). Cannabis Consumption in People Living with HIV: Reasons for Use, Secondary Effects, and Opportunities for Health Education. Cannabis Cannabinoid Research, 4(3), 204–213. 10.1089/can.2018.0068 31579835 PMC6757238

[bibr15-00914509231183147] Crescenzi N. (2019, July 6). Victoria cannabis compassion club closes its doors after 20 years in operation. Victoria News. https://www.vicnews.com/news/victoria-cannabis-compassion-club-closes-its-doors-after-20-years-in-operation/

[bibr16-00914509231183147] DeAngelo S. (2015). The cannabis manifesto: A new paradigm for wellness. North Atlantic Books.

[bibr17-00914509231183147] Decorte T. (2015). Cannabis social clubs in Belgium: Organizational strengths and weaknesses, and threats to the model. International Journal of Drug Policy, 26(2), 122–130. 10.1016/j.drugpo.2014.07.016 25179934

[bibr18-00914509231183147] Decorte T. Pardal M. (2020). Insights for the design of Cannabis social club regulation. In Decorte T. Lenton S. Wilkins C. (Eds.), Legalizing cannabis: Experiences, lessons and scenarios (pp. 409–426). Routledge.

[bibr19-00914509231183147] Decorte T. Pardal M. Queirolo R. Boidi M. F. Avilés C.S. Franquero Parés Ò. (2017). Regulating cannabis social clubs: A comparative analysis of legal and self-regulatory practices in Spain, Belgium and Uruguay. International Journal of Drug Policy, 43, 44–56. 10.1016/j.drugpo.2016.12.020 28189980

[bibr20-00914509231183147] Egan-Elliott R. (2023, March 31). Victoria Cannabis Buyers Club club to sue governments after raid. Times Colonist. https://www.timescolonist.com/local-news/victoria-cannabis-buyers-club-club-to-sue-governments-after-raid-6786780

[bibr21-00914509231183147] Feldman H. W. Mandel J. (1998). Providing medical marijuana: The importance of cannabis clubs. Journal of Psychoactive Drugs, 30(2), 179–186. 10.1080/02791072.1998.10399688 9692380

[bibr22-00914509231183147] Fischer B. Kuganesan S. Room R. (2015). Medical marijuana programs: Implications for cannabis control policy – observations from Canada. International Journal of Drug Policy, 26(1), 15–19. 10.1016/j.drugpo.2014.09.007 25287942

[bibr23-00914509231183147] Fischer B. Rusell C. Boyd N. (2020). A century of cannabis control in Canada. In Decorte T. Lenton S. Wilkins C. (Eds.), Legalizing cannabis: Experiences, lessons and scenarios (pp. 89–115). Routledge.

[bibr25-00914509231183147] Gagnon M. Gudiño D. Guta A. Strike C. (2020). What can we learn from the English-language media coverage of cannabis legalization in Canada? Substance Use and Misuse, 55(8), 1378–1381. 10.1080/10826084.2020.1741639 32204651

[bibr26-00914509231183147] Gehring N. D. Speed K. A. Launier K. O'Brien D. Campbell S. Hyshka E. (2022). The state of science on including inhalation within supervised consumption services: A scoping review of academic and grey literature. International Journal of Drug Policy, 102, 103589. 10.1016/j.drugpo.2022.103589 35101668

[bibr27-00914509231183147] Hager M. Imam H. (2020, February 11). The writing is on the wall for Vancouver’s handful of illegal cannabis shops. Globe and Mail. https://www.theglobeandmail.com/canada/british-columbia/article-the-writing-is-on-the-wall-for-vancouvers-handful-of-illegal-cannabis/

[bibr28-00914509231183147] Hathaway A. D. Rossiter K. (2007). Medical marijuana, community building, and Canada's compassionate societies. Contemporary Justice Review, 10(3), 283–296. 10.1080/10282580701526088

[bibr29-00914509231183147] Health Canada (2016). A framework for the legalization and regulation of cannabis in Canada: The finale report of the taskforce on cannabis legalization and regulation. https://healthycanadians.gc.ca/task-force-marijuana-groupe-etude/framework-cadre/alt/framework-cadre-eng.pdf

[bibr30-00914509231183147] Hlatshwako T. G. Shah S. J. Kosana P. Adebayo E. Hendriks J. Larsson E. C. Hensel D. J. Erausquin J. T. Marks M. Michielsen K. Saltis H. Francis J. M. Wouters E. Tucker J. D. (2021). Online health survey research during COVID-19. Lancet Digit Health, 3(2), e76–e77. 10.1016/S2589-7500(21)00002-9 33509387 PMC10000261

[bibr31-00914509231183147] Jansseune L. Pardal M. Decorte T. Franquero Parés Ò (2019). Revisiting the birthplace of the cannabis social club model and the role played by cannabis social club federations. Journal of Drug Issues, 49(2), 338–354. 10.1177/0022042618815690

[bibr32-00914509231183147] Kittel J. (2023). VCBC reaches out to premiere after 3rd raid. https://cannabisdigest.ca/vcbc-reaches-out-to-premiere-after-3rd-raid/

[bibr34-00914509231183147] Krause P. Szekely O. Bloom M. Christia F. Daly S. Lawson C. Marks Z. Milliff A. Miura K. Nielson R. Reno W. Souleimanov E. A. Zakayo A. (2021). COVID-19 and fieldwork: Challenges and solutions. PS: Political Science & Politics, 54(2), 264–269. 10.1017/S1049096520001754

[bibr35-00914509231183147] Lake S. Walsh Z. Kerr T. Cooper Z. D. Buxton J. Wood E. Ware M. A. Milloy M.-J. (2019). Frequency of cannabis and illicit opioid use among people who use drugs and report chronic pain: A longitudinal analysis. PLoS Medecine, 16(11), e1002967. 10.1371/journal.pmed.1002967 PMC6863529 31743343

[bibr36-00914509231183147] Lavallée P. Beaumont J.-F. (2015). Why we should put some weight on weights. Survey insights: Methods from the field, weighting: Practical issues and ‘how to’ approach. https://surveyinsights.org/?p=6255

[bibr37-00914509231183147] Lucas P. (2009). Moral regulation and the presumption of guilt in Health Canada’s medical cannabis policy and practice. International Journal of Drug Policy, 20(4), 296–303. 10.1016/j.drugpo.2008.09.007 19124233

[bibr38-00914509231183147] Lucas P. (2012). It can't hurt to ask; A patient-centered quality of service assessment of health Canada's medical cannabis policy and program. Harm Reduction Journal, 9(2), 1–11. 10.1186/1477-7517-9-2 22214382 PMC3285527

[bibr39-00914509231183147] Lucas P. Boyd S. Molly M.-J. Walsh Z. (2021). Cannabis significantly reduces the use of prescription opioids and improves quality of life in authorized patients: Results of a large prospective study. Pain Medecine, 22(3), 727–739. 10.1093/pm/pnaa396 PMC7971472 33367882

[bibr40-00914509231183147] Lucas P. G. (2008). Regulating compassion: An overview of Canada's federal medical cannabis policy and practice. Harm Reduction Journal, 5(1), 5. 10.1186/1477-7517-5-5 18226254 PMC2267789

[bibr41-00914509231183147] Malott M. (2009). Medical marijuana: The story of Dennis Peron, the San Fransisco Cannabis Buyers Club, and the ensuing road to decriminalization.

[bibr42-00914509231183147] Milloy M.-J. Gagnon M. (2022). Cannabis could be safe alternative to toxic drug supply, so why is access being cut off? https://www.straight.com/news/420-cannabis-could-be-safe-alternative-to-toxic-drug-supply-so-why-is-access-being-cut-off

[bibr43-00914509231183147] Ministry of Public Safety and Solicitor General (2022a). Non-medical cannabis consumption space engagement. https://engage.gov.bc.ca/app/uploads/sites/741/2022/04/8711_Cannabis_Consumption_Discussion_Paper_PSSG_v5.pdf

[bibr44-00914509231183147] Ministry of Public Safety and Solicitor General (2022b). What we heard: Cannabis consumption spaces public engagement. https://engage.gov.bc.ca/govtogetherbc/impact/cannabis_consumption_spaces_results/

[bibr45-00914509231183147] Mok J. Milloy M.-J. Grant C. Lake S. DeBeck K. Hayashi K. Socías M. E. (2021). Use of Cannabis for harm reduction among people at high risk for overdose in Vancouver, Canada (2016–2018). American Journal of Public Health, 111(5), 969–972. 10.2105/AJPH.2021.306168 33734849 PMC8033988

[bibr46-00914509231183147] Ng J. Y. Homayouni P. Usman S. Gomes Z. (2022). The medical cannabis regulatory framework in Canada: A narrative review. European Journal of Integrated Medicine, 50, 102104. 10.1016/j.eujim.2022.102104

[bibr47-00914509231183147] Obradors-Pineda A. Bouso J.-C. Parés-Franquero Ò Romani J.-O. (2021). Harm reduction and cannabis social clubs: Exploring their true potential. International Journal of Drug Policy, 97, 1–4. 10.1016/j.drugpo.2021.103358 34252786

[bibr48-00914509231183147] Pardal M. (2016). Cannabis social clubs through the lens of the drug user movement. Tijdschrift Over Cultuur En Criminaliteit, 6(2), 47–58. 10.5553/TCC/221195072016006002003

[bibr50-00914509231183147] Pardal M. Decorte T. Bone M. Parés O. Johansson J. (2022). Mapping cannabis social clubs in Europe. European Journal of Criminology, 19(5), 1–24. 10.1177/1477370820941392

[bibr49-00914509231183147] Pardal M. (2023). The cannabis social club. Routledge.

[bibr51-00914509231183147] Parés-Franquero Ò Jubert-Cortiella X. Olivares-Gálvez S. Díaz-Castellano A. Jiménez-Garrido D. F. Bouso J. C. (2019). Use and habits of the protagonists of the story: Cannabis social clubs in Barcelona. Journal of Drug Issue, 49(4), 607–624. 10.1177/0022042619852780

[bibr52-00914509231183147] Queirolo R. Boidi M. F. Cruz J. M. (2016). Cannabis clubs in Uruguay: The challenges of regulation. International Journal of Drug Policy, 34, 41–48. 10.1016/j.drugpo.2016.05.015 27475713

[bibr53-00914509231183147] Reddon H. DeBeck K. Socías M. E. Lake S. Dong H. Karamouzian M. Hayashi K. Kerr T. Milloy M.-J. (2020). Frequent Cannabis use and cessation of injection of opioids, Vancouver, Canada, 2005–2018. American Journal of Public Health, 110(10), 1553–1560. 10.2105/AJPH.2020.305825 32816538 PMC7483125

[bibr54-00914509231183147] Reiman A. (2014). Cannabis distribution: Coffee shops to dispensaries. In Pertwee R R. (Ed.), Handbook of cannabis (pp. 339–355). Oxford University Press.

[bibr55-00914509231183147] Rudisuela J. (2021, March 25). Raids and an eviction notice won't be the end of Victoria Cannabis Buyers Club. Capital Daily. https://www.capitaldaily.ca/news/victoria-cannabis-buyers-club

[bibr56-00914509231183147] R. v. Smith. 2015 SCC 34 (CanLII), [2015] 2 SCR 602, https://canlii.ca/t/gjgtl

[bibr57-00914509231183147] Sevelius J. M. Gutierrez-Mock L. Zamudio-Haas S. McCree B. Ngo A. Jackson A. Clynes C. Venegas L. Salinas A. Herrera C. Stein E. Operario D. Gamarel K. (2020). Research with marginalized communities: Challenges to continuity during the COVID-19 pandemic. AIDS Behavior, 24(7), 2009–2012. 10.1007/s10461-020-02920-3 32415617 PMC7228861

[bibr58-00914509231183147] Shinew K. Gibson H. Schneider I. Wynveen C. Hendricks W. Budruk M. Farrell E. (2022). Reflections on conducting research in uncertain times. Leisure Studies, 41(3), 446–454. 10.1080/02614367.2021.2022181

[bibr60-00914509231183147] Smith T. (2016). Victoria Council issues cannabis buyers club smoking exemption. http://www.hempology.ca/2016/11/30/victoria-council-issues-cannabis-buyers-club-smoking-exemption.

[bibr61-00914509231183147] Smith T. Kettel J. (2019). Victoria Cannabis Buyers Club: Strategic Plan 1.0. https://cannabisdigest.ca/the-vcbc-strategic-plan-goes-public/

[bibr62-00914509231183147] Socías M. E. Choi J. Lake S. Wood E. Valleriani J. Hayashi K. Kerr T. Milloy M.-J. (2021). Cannabis use is associated with reduced risk of exposure to fentanyl among people on opioid agonist therapy during a community-wide overdose crisis. Drug and Alcohol Dependency, 219, 108420. 10.1016/j.drugalcdep.2020.108420 PMC8006801 33342591

[bibr63-00914509231183147] Sorensen J. L. Drannen J. Shingle M. (2021). Legalization of cannabis in Canada-local media analysis. American Journal of Addiction, 31(2), 148–151. 10.1111/ajad.13263 35102629

[bibr64-00914509231183147] Stake R. (1995). The art of case study research. Sage.

[bibr65-00914509231183147] Stake R. (2005). Qualitative case studies. In Denzin N. K. Lincoln Y. S. (Eds.), The sage handbook of qualitative research (pp. 443–466). Sage.

[bibr66-00914509231183147] St.Pierre M. Daniels S. Walsh Z. (2022). Cannabis substitution: The Canadian experience. In Hathaway A. D. Smith McCann C. J. (Eds.), The high north: Cannabis in Canada (pp. 146–163). UBC Press.

[bibr67-00914509231183147] Subritzky T. (2018). Beyond deficit and harm reduction: Incorporating the spectrum of wellness as an interpretive framework for cannabis consumption. International Journal of Drug Policy, 60, 18–23. 10.1016/j.drugpo.2018.07.013 30086481

[bibr68-00914509231183147] Thrane C. (2022). Doing statistical analysis : A student’s guide to quantitative research. Taylor & Francis Group.

[bibr69-00914509231183147] Valleriani J. (2022). Medical cannabis dispensaries: A conduit for change? In Hathaway A. D. Smith McCann C. J. (Eds.), The high north: Cannabis in Canada (pp. 97–110). UBC Press.

[bibr70-00914509231183147] Valleriani J. Haines-Saah R. Capler R. Bluthenthal R. Socias E. M. Milloy M.-J. Kerr T. McNeil R. (2020). The emergence of innovative cannabis distribution projects in the downtown eastside of Vancouver, Canada. International Journal of Drug Policy, 79, 102737. 10.1016/j.drugpo.2020.102737 32289590 PMC7308205

[bibr71-00914509231183147] Victoria News Staff (2023, February 21). Victoria cannabis club moving to new North Park location after 22 years. Victoria News. https://www.vicnews.com/business/victoria-cannabis-club-moving-to-new-north-park-location-after-22-years/

[bibr72-00914509231183147] Voon P. Greer A. M. Amlani A. Newman C. Burmeister C. Buston J. (2018). Pain as a risk factor for substance use: A qualitative study of people who use drugs in British Columbia, Canada. Harm Reduction Journal, 15, 35. 10.1186/s12954-018-0241-y 29976203 PMC6034304

[bibr73-00914509231183147] Walsh Z. Callaway R. Belle-Isle L. Capler R. Kay R. Lucas P. Holtzman S. (2013). Cannabis for therapeutic purposes: Patient characteristics, access, and reasons for use. International Journal of Drug Policy, 24(6), 511–516. 10.1016/j.drugpo.2013.08.010 24095000

[bibr74-00914509231183147] Watson T. M. Hyshka E. Bonato S. Rueda S. (2019). Early-stage cannabis regulatory policy planning across Canada's four largest provinces: A descriptive overview. Substance Use and Misuse, 54(10), 1691–1704. 10.1080/10826084.2019.1608249 31076006

[bibr75-00914509231183147] Wyton M. (2022, April 21). ‘We’re saving lives, and being punished for doing it’. The Tyee. https://thetyee.ca/News/2022/04/21/Cannabis-Club-Saving-Lives-Punished-Doing-It/

